# Protocol for Chinese lung cancer evolution and microenvironment tracking under therapy study

**DOI:** 10.1515/jtim-2025-0047

**Published:** 2025-12-12

**Authors:** Wenxiang Wang, Yue He, Xiaoqiu Yuan, Lin Weng, Jing Bai, Xu Liu, Li Han, Junnan Xu, Shuai Wang, Shanshan Lu, Jian Bai, Hui Quan, Qingyun Liu, Xinrui Li, Yuxuan Xiao, Yun Wang, Qingna Zhang, Na Zhou, Yanyan Hou, Hao Li, Yun Li, Fan Yang, Jun Wang, Kezhong Chen, The CLEVER Consortium

**Affiliations:** Thoracic Oncology Institute, Peking University People's Hospital, Beijing, China; Research Unit of Intelligence Diagnosis and Treatment in Early Non-small Cell Lung Cancer, Chinese Academy of Medical Sciences, 2021 RU002, Peking University People's Hospital, Beijing, China; Department of Thoracic Surgery, Peking University People's Hospital, Beijing, China; Department of Pathology, Peking University People's Hospital, Beijing, China; Kanghui Biotech Co., Ltd., Liaoning, Shenyang Province, China; College of Future Technology, Peking University, No. 5 Yiheyuan Road, Beijing, China; Geneplus-Beijing Institute, No. 6 Building, Peking University Medical Industrial Park, Zhongguancun Life Science Park, Beijing, China; Annoroad Gene Technology Co., Ltd, Beijing, China; Institute of Advanced Clinical Medicine, Peking University, Beijing, China

**Keywords:** lung cancer, tumor heterogeneity, cancer evolution

## Abstract

**Background and objectives:**

Recent molecular landscape studies characterizing lung cancer mostly utilize single tumor regions, ignoring tumor heterogeneity and evolutionary patterns. To address this issue, the TRAcking NSCLC Evolution through therapy (TRACERx) Consortium established a multiregion sampling of Western lung cancer to investigate its longitudinal evolutionary dynamics. Due to ancestral differences, there are still major gaps in understanding oriental lung cancer.

**Methods:**

We have established a prospective cohort of patients with non-small cell lung cancer (NSCLC) named Chinese lung cancer evolution and microenvironment tracking under therapy (CLEVER) study.

**Discussion:**

By acquiring comprehensive genetic variation data and clonal events from multiple regions of the tumor, we aim to decipher the molecular evolution code of Chinese lung cancer. The analysis of cell composition and spatial structure further characterizes the remodeling effects of tumor evolution and neoadjuvant therapy on the tumor microenvironment, helping to identify important markers that influence treatment effectiveness and recurrence. By monitoring mutations using a personalized minimal residual disease (MRD) panel from tumors at baseline, we can trace evolutionary significance and clonality, aiding in therapy decisionmaking. The CLEVER study significantly contributes to the development of personalized treatment strategies for lung cancer patients, enhancing outcomes and tailoring interventions to individual patient profiles.

## Introduction

Lung cancer, with non-small cell lung cancer (NSCLC) accounting for 85% of cases, stands as the malignancy with the highest mortality worldwide.^[1]^ An increasing body of cancer research has led to an unprecedented view of the molecular landscape of lung cancer throug next generation sequencing techniques, including DNA sequencing and RNA sequencing.^[2,3]^ Tumor evolution, as a key concept in understanding the onset and progression of cancer, not only contributes to tumor heterogeneity but also results in individual treatment differences. Through multi-region sampling and advanced sequencing technologies, researchers can uncover the evolutionary trajectories of tumors across different temporal and spatial scales. As the largest study of the evolution of NSCLC to date, the TRAcking NSCLC Evolution through therapy [Rx] (TRACERx) study employs multi-region sampling to investigate various aspects of lung cancer, including clonal evolution, genomic instability, and intratumoral heterogeneity to identify prognostic biomarkers that can inform treatment strategies and improve patient outcomes.^[4, 5]^ However, this study primarily involves patients of Western ancestry, which means it provides valuable insights into the biological mechanisms and progression of lung cancer within this specific population.

Lung cancer has significant racial differences, with significant variations in incidence, progression, and outcomes across different ethnic groups.^[6]^ These disparities are influenced by a complex interplay of genetic, environmental, and lifestyle factors. Studies have shown that genetic mutations, such as those in the EGFR and ALK genes, occur at different frequencies in Eastern and Western populations, leading to variations in treatment responses and prognoses.^[7, 8]^ Furthermore, a study comparing 305 cases of lung adenocarcinoma in individuals of East Asian ancestry with 249 cases in individuals of European ancestry found that the East Asian group exhibited more stable genome, characterized by fewer mutations and copy number changes. This finding highlighted important ancestry differences in lung cancer between Eastern and Western populations.^[9]^ However, for the eastern lung cancer multi-region lung cancer study, the explorations were limited due to the small sample size.^[10, 11]^ To sum up, it is necessary and urgent to establish a large multi-region sampling cohort of Chinese lung cancer population.

In our preliminary retrospective investigation of 81 patients with stage I NSCLC (Chinese PKUPH cohort, named CHN-P), we observed that there are strong ancestry disparities in lung cancer and Asian NSCLCs exhibit diverse genomic features. Regrettably, only a small number of patients, all of whom were at stage I, and the multi-region sampling methods were not consistent.^[12]^ To deepen our understanding of intratumoral heterogeneity in Chinese lung cancer patients, we expanded our study to include patients with stage I-IIIA NSCLC and implemented a standardized multi-region sampling process. This approach not only allowed us to explore the molecular signatures and clonal evolution inside tumors but also enabled us to investigate the immune microenvironment in the marginal zone and normal lung tissue. By uncovering the evolutionary molecular mechanisms and changes in the immune microenvironment from normal to aggressive borderline to tumor, we aim to provide valuable insights for clinical diagnosis and treatment strategies of lung cancer. With the prevalence of neoadjuvant therapy and its limited clinical benefits, there is a growing interest in identifying biomarkers and immune characteristics that are related to therapeutic efficacy. Hu *et al*. mapped tumor microenvironment changes during neoadjuvant immunotherapy by utilizing single-cell sequencing on tumor and peripheral blood samples and identified FCRL4+FCRL5^+^ B cells, CD 16^+^CX3CR1^+^ monocytes, and plasma estrogen changes as novel biomarkers.^[13]^ Xia *et al*. found a positive correlation between TYROBP expression and favorable responses to neoadjuvant camrelizumab, which was associated with increased infiltration of CD8^+^ T cells and enhanced effector responses of natural killercells.^[14]^ However, there have been no studies employing multi-region sampling cohorts for multi-omics research to explore the association of tumor heterogeneity with neoadjuvant efficacy and long-term survival. This study addresses this gap by innovatively collecting multi-region samples post-neoadjuvant therapy to investigate spatial regression patterns of tumors and changes in intratumoral heterogeneity. Furthermore, we have integrated various advanced spatial omics technologies to gain a more comprehensive understanding of the molecular landscape under neoadjuvant therapy.

The Chinese lung cancer evolution and microenvironment tracking under therapy (CLEVER) cohort study is a prospective single-center lung cancer multi-region sample cohort. This study examines how tumor evolution and intratumoral heterogeneity influence cancer treatment, outcomes, and prognosis. This protocol outlines the procedures for the CLEVER study (Version 1) and was prepared in accordance with the SPIRIT reporting guidelines.^[15]^

## Study design and highlights

**Figure 1:**
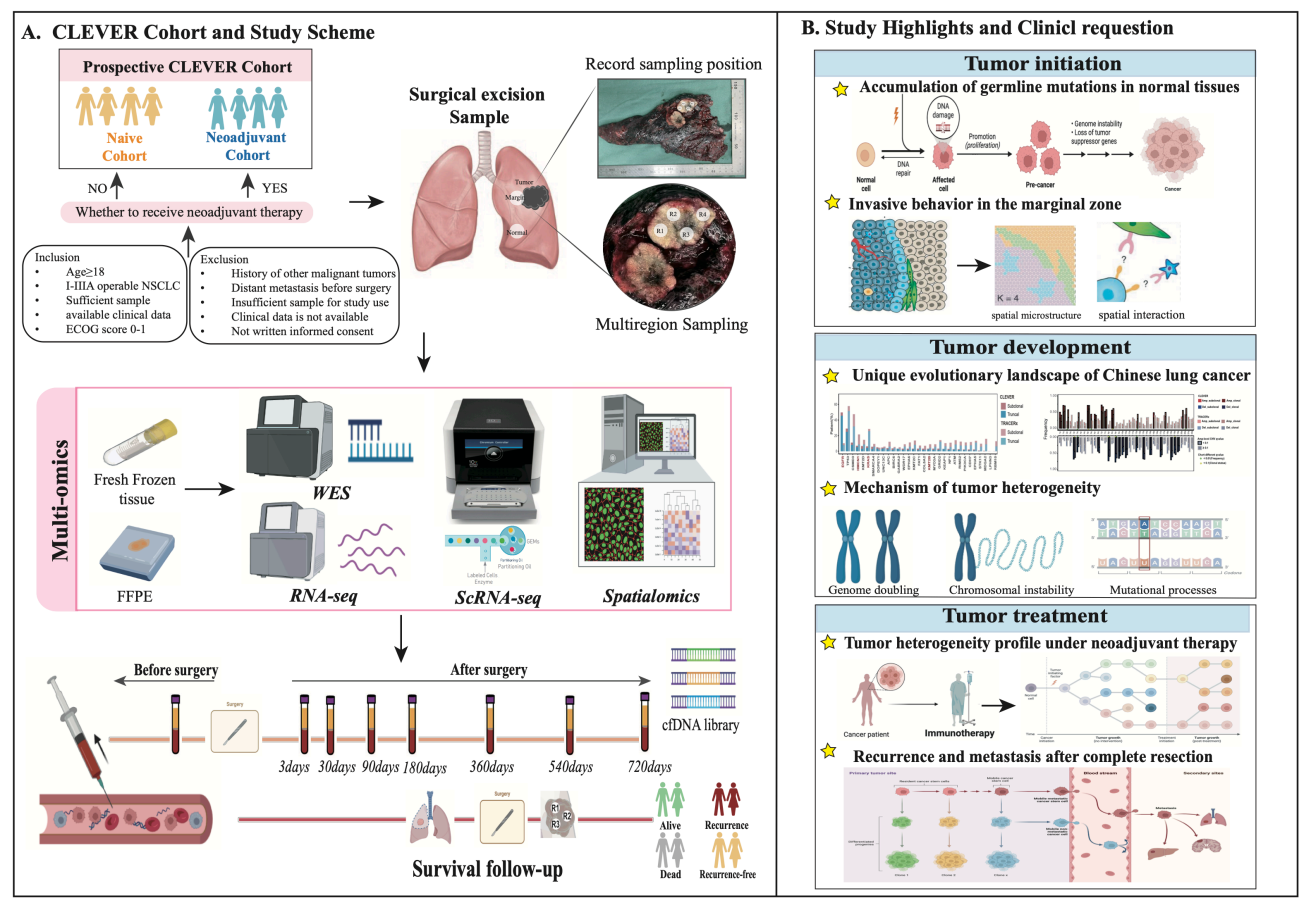
Overview of CLEVER study. (A) Study design and scheme. (B) Key questions and highlights of this study. Image created by BioRender. com, with permission. CLEVER: Chinese Lung cancer EVolution and microEnvironment tracking under theRapy; FFPE: formalin fixation and paraffin embedding; WES: whole-exome sequencing; RNA-seq: RNA sequencing; ScRNA-seq: single cell RNA sequencing.

We conduct a prospective large-scale multi-region Chinese lung cancer cohort called CLEVER study from Peking University People’s Hospital. The cohort is divided into two subcohorts: the naïve cohort, which includes patients who have not received neoadjuvant therapy before surgery, and the neoadjuvant cohort, which includes those who have. During surgery, samples of tumor tissue, marginal tissue, and matched normal tissue are collected. These samples undergo comprehensive multidimensional omics analyses, including whole exome sequencing (WES), RNA sequencing (RNA-seq), single-cell sequencing, and multiple spatial omics techniques such as Stereo-seq, digital spatial profiling (DSP), and imaging mass cytometry (IMC). Additionally, preoperative blood samples are collected on the morning of the surgery, and postoperative blood samples are collected during a two-year follow-up period for longitudinal dynamic monitoring of minimal residual disease (MRD) (Figure 1A). The primary endpoint of the study are two-year disease-free survival (DFS) and overall survival (OS). The secondary endpoint is to identify available predictors of clinical benefit.

The CLEVER study aims to comprehensively characterize tumor evolution and immune microenvironment profiles of Chinese NSCLC, establishing accurate prognostic machine learning model and dynamic longitudinal MRD recurrence monitoring system. It investigates the entire process of tumor initiation, development, and treatment, addressing several key unresolved questions: (1) mutation accumulation and clonal evolution of normal lung tissue; (2) invasive molecular behaviors and microenvironment characteristics at the tumor-normal tissue interface; (3) unique evolutionary events and molecular profiles of Eastern lung cancer compared to Western lung cancer; (4) mechanism of tumor heterogeneity and ethnic differences; (5) the heterogeneous molecular landscape of lung cancer under neoadjuvant therapy; and (6) clinically available biomarkers of recurrence and metastasis. Through multi-site sampling and multi-omics analysis, this study aims to provide a deeper and clearer understanding of these questions, which constitutes a significant highlight of the research (Figure 1B).

## Patients and samples

### Ethics approval and consent to participate

The CLEVER study has received approval from the Ethics Committee of Peking University People’s Hospital (approval number 2022PHB140-001). It is registered with ClinicalTrials. gov under the identifier NCT05352035. Patient recruitment for the study began in April 2022.

### Patients enrollment

#### Sample size calculation

The CLEVER study aims to recruit over 1000 patients to investigate the molecular evolution patterns of lung cancer in the Chinese population. It is anticipated that the proportion of recurrence events by the end of the follow-up period will range from 15% to 30%. We have calculated the sample size for the first phase of CLEVER through the following three considerations.

##### Consideration for survival analysis

In the first phase of the CLEVER study, we aim to compare 2-year DFS between predefined high-risk and low-risk groups using a two-sided log-rank test at α = 0.05 and 80% power (1-β = 0.80). Based on our preliminary data (CHN-P cohort),^[12]^ we assume a 2-year DFS event (recurrence or death) rate of 25% in the overall population. We further posit that the high-risk group will experience a hazard ratio (HR) of 2.0 relative to the low-risk group.

Applying the Freedman formula for survival comparisons:^[16]^


$$N_{\text{events }}=\frac{{\left(Z_{1-\frac{\alpha }{2}}+Z_{1-\beta }\right)}^{2}}{(\ln HR{)}^{2}}=\frac{(1.96+0.84{)}^{2}}{(\ln2{)}^{2}}\approx 43\text{ events }$$

Assuming a 25% event rate, at least 172 patients are needed to observe the required number of DFS events.

##### Consideration for recurrence prediction validation

In this study, we aim to validate a machine-learning-based lung cancer recurrence prediction model with anticipated sensitivity of 90% ± 10% and specificity of 90% ± 10%. To calculate the necessary sample size for this diagnostic validation, we anchor our calculations to a fixed two-year recurrence (event) rate of 20%, based on retrospective study of recurrence after complete resection of early NSCLC.^[17, 18]^

Using Buderer’s formula for sensitivity and specificity sample size estimation:^[19, 20]^


$$N=\frac{Z^{2}\cdot p(1-p)}{d^{2}\cdot \text{ Prevalence }}=\frac{(1.96{)}^{2}\cdot 0.9\cdot 0.1}{0.1^{2}\cdot 0.2}\approx 173\text{ patients }$$

Here p is the anticipated sensitivity or specificity (0.90), d is the half-width of the 95% confidence interval (0.1), and Prevalence is the event prevalence (0.25). This calculation yields approximately 173 patients required to estimate sensitivity and specificity with ±10% precision at α = 0.05.

##### Consideration for machine learning modeling

The novel lung cancer recurrence prediction model will be constructed based on machine-learning algorithms (section 5.2). For the model development, we adopt a ratio of 6:2:2 for the training, validation, and test sets. This division ratio is based on the consideration that 60% training provides adequate data for model fitting and feature selection, while 20% validation allows unbiased hyperparameter tuning, and 20% test ensures a fully held-out assessment to guard against overfitting. This ratio balances model complexity and evaluation rigor, as recommended for medium-sized cohorts.^[21, 22, 23]^ When 173 patients are recruited for statistical power in the training set, a total of 289 cases are required.

In summary, to address the above three dimensions of statistical consideration and the disruption caused by loss to follow-up, we plan to enroll 300 patients in the first phase. This provides sufficient power for survival analysis while also accommodating downstream modeling requirements.

#### Inclusion and exclusion criteria

##### Inclusion Criteria

(1) Age ≥ 18 years old; (2) Stage I-IIIA operable NSCLC (ninth AJCC); (3) Sufficient tumor tissue and blood sample with a suspected tumor with a short diameter of at least 15 mm on the preoperative image; (4) NSCLC was confirmed by histopathology, and clinical, imaging and follow-up data were available; (5) Eastern Cooperative Oncology Group (ECOG) Performance Status of 0–1; (6) Written informed consent.

#### Exclusion Criteria

(1) Any other current malignancy or malignancy diagnosed or relapsed within the last 5 years; (2) Evidence of distant metastasis before surgery or inoperable state; (3) Infectious diseases such as HIV, hepatitis B, hepatitis C or syphilis; (4) Insufficient tumor tissue or blood sample; (5) Clinical-pathologic data, imaging data, or follow-up data are not available.

#### Patient management

In the CLEVER study, all eligible patients are presented at a weekly multidisciplinary tumor board meeting. Decisions regarding neoadjuvant therapy versus upfront surgery are made by consensus based on published authoritative guidelines, taking into account tumor stage (II–IIIA generally considered for neoadjuvant), resectability, and patient preference. Once the treatment plan is determined, research staff obtain written informed consent from participants in the hospital room. The following two questions are explained in detail to ensure that patients fully understand. First, this study involves repeated blood sampling of eight scheduled time points over two years. Potential risks (*e.g*., transient pain, bruising, hematoma, infection) are described, and standard phlebotomy precautions are assured. Second, this study involves a two-year follow-up schedule, including clinical visits and imaging studies. Patients are informed of data confidentiality measures and their right to withdraw at any time without affecting clinical care. Patients treated with direct surgical resection belongs to the Naïve cohort while those undergoing surgical resection following neoadjuvant immunotherapy belongs to the Neoadjuvant cohort). For patients assigned to the neoadjuvant cohort, the consent form includes additional sections that explain the risk and benefit from the neoadjuvant therapies. Participants have the opportunity to ask questions and are provided contact information for study coordinators and the ethics committee. Comprehensive clinical information is collected from all participants, including age, gender, smoking history, radiological image characteristics, and histopathology descriptions.

### Sample collection

#### Tissue samples

In order to accurately sample and record the position, we have developed a standardized multi-region sampling process. Initially, the surgical sample is dissected along its maximum diameter. Equal-sized samples are then taken from one side of the tumor using a skin perforator. For smaller tumors, consider sampling from various locations on the opposite side. Samples from at least two tumor regions must be separated by 0.3 cm to 1 cm, depending on the tumor size, for inclusion in the study. Border samples should be collected at the interface between tumor and normal tissue to ensure that both tumor and normal samples are obtained. Paired normal tissue samples should be taken at least 2 cm away from the tumor edge. Last but not least, the central coordinates of each sampling point were recorded using a double-scale square and documented with photographs.

All tissue samples must be immediately placed into freezing tubes for preservation at -80°C. If the tumor is sufficiently large, a set of paraffin-embedded samples should also be collected for all tissues.

#### Blood samples

Participants in the study will have their blood drawn at eight prespecified time points: pre-surgery, 3 days after surgery, 1 month after surgery, 3 months after surgery, 6 months after surgery, 12 months after surgery, 18 months after surgery, and 24 months after surgery. Preoperative blood is collected on the morning of the surgery, while postoperative blood is collected according to the study design time points. As the follow-up blood collection time may have a time deviation from the set blood collection point, when recording the blood collection follow-up, we will truthfully record the blood collection time of that day and further calculate which time point the blood sample belongs to. We allow a maximum deviation of ± 1 month around each scheduled postoperative draw to accommodate clinical logistics. Sensitivity analyses will be performed by excluding samples collected outside the ± 2-week window and re-estimating MRD dynamics and their correlation with outcomes, to confirm the robustness of temporal patterns. For patients receiving neoadjuvant therapy, blood samples will also be taken on the first day of each course of treatment.

Peripheral blood samples, approximately 20 mL each, are collected into Cell-Free DNA BCT tubes (Streck, Omaha, USA) and transported to the laboratory. Plasma and white cell samples are separated according to the provided instructions within 24 h and stored at -80°C.

### Data collection and management

According to the medical record numbers of enrolled patients, clinical, imaging, and pathological data were completely, correctly, and clearly entered into the statistical information table through medical record inquiry. Clinical data are archived and stored as a database in numbered order, with multiple backups to ensure proper storage. The original files are retained for the period specified by relevant guidelines. Original data are managed by designated personnel and stored on the hospital’s internal computer hard disk, which is not accessible from external networks. The final archived data of this study are stored only in serial numbers and do not contain any personal privacy information of patients. No patient privacy information was involved when the data were used for publication.

## Multi-omics profiling

### Nucleic acid extraction

Genomic DNA and total RNA are purified from the frozen tumor and normal tissues using the AllPrep DNA/RNA Mini Kit (Qiagen, Hilden, Germany). DNA in paired white cell samples is extracted using the TIANamp Genomic DNA Kit (Tiangen, Beijing, China). Cell-free DNA in the plasma samples is extracted using the QIAamp Circulating Nucleic Acid Kit (Qiagen, Hilden, Germany). Nucleic acid concentrations are quantified using either the Qubit HS dsDNA Kit or the Qubit RNA HS Assay Kit (Invitrogen, Carlsbad, USA).

### Whole exome sequencing and analysis

The DNA library is prepared with an input of 200 ng genomic DNA using the VAHTS Universal Plus DNA Library Prep Kit for Illumina V2. The subsequent hybridization capture is performed using the SureSelect XT target enrichment system. The final DNA library is quantified using the Qubit 3.0 and fragment detection is conducted with the Agilent 2100. Finally, the DNA library is sequenced using paired-end 150 bp reads on the Illumina NovaSeq 6000 platform.

The sequencing data is analyzed using the WES pipeline. The sequencing data from white cells is used to exclude germline mutations. Clean reads in FASTQ format are aligned to the human UCSC reference genome (hg19/GRCh37) using the Burrows-Wheeler Aligner (BWA, v0.7.15). Sambamba (v0.6.8) is used to process PCR duplicates in the mapped BAM files. The Genome Analysis Toolkit (GATK v4.0.11.0) is employed for local realignment and base quality recalibration to compute sequencing coverage and depth. Single nucleotide variations (SNVs) and small insertions and deletions (Indels: < 50 bp) are identified using GATK MuTect2 (v1.1.4). Multiple testing correction for gene-level mutation frequency comparisons will be performed using the Benjamini-Hochberg procedure, with an adjusted p-value (FDR) cutoff of < 0.05.

Based on multi-region sequencing, region-based tumor mutation calling is combined to recall tumor-informed mutations, allowing for the acquisition of new recalled mutations for each region. Using these recalled region-level mutations, the phylogenetic tree is reconstructed using the CNIPHER method,^[24]^ enabling the identification of clonal and subclonal mutations. Somatic copy number alterations (SCNAs) are processed using ASCAT (V2.3), which allows for the determination of intratumoral heterogeneity in both SNVs and SCNAs, specifically assessing subclonal mutation fractions and subclonal SCNAs fractions. This comprehensive analysis facilitates a deep understanding of evolutionary events within the tumor, characterized by intratumoral heterogeneity, clonal illusion, subclonal expansion, whole genome doubling, weighted genome integrity index (wGII), and loss of heterozygosity (LOH). These insights provide a detailed depiction of the tumor’s evolutionary landscape and its underlying genetic dynamics.

### RNA sequencing and analysis

To construct a transcriptome sequencing library, 1–3 μg of total RNA is prepared using the VAHTS Universal V6 RNA-Seq Library Prep Kit for Illumina (NR604-01/02). The final RNA library is quantified using the Qubit 3.0, and fragment analysis is conducted with the Agilent 2100. Additionally, the Bio-RAD CFX 96 fluorescence quantitative PCR is used to quantify the effective concentration of the RNA library (ensuring it is greater than 10 nmol/L) using the Bio-RAD KIT iQ SYBR Green. The RNA library is then sequenced using paired-end 150 bp reads on the Illumina NovaSeq 6000 platform.

The sequencing data is aligned to the human UCSC reference genome (hg19/GRCh37) using the STAR aligner (version 2.7.4a) and annotated with ANNOVAR software. Subsequent analyses include immune cell infiltration assessment, identification of differentially expressed genes (DEGs), enrichment analysis, and subtypes clustering. DEGs will be identified with DESeq2, applying the Benjamini–Hochberg FDR correction. Genes with FDR < 0.05 and |log₂FoldChange| > 1 will be considered significant. These analyses aim to explore the heterogeneous tumor microenvironment, providing insights into the complex interactions and variations within the tumor.

### Single-cell RNA-seq sequencing

Fresh or frozen tissue samples are enzymatically digested to obtain single-cell suspensions, achieving a concentration of 700–1200 living cells per ml. Libraries are prepared using the Chromium Next GEM Single Cell 3’ Reagent Kits v3.1, following the manufacturer’s protocol. The purified library is sequenced on the Illumina NovaSeq 6000 platform with a sequencing length of 150 bases in a paired-end format.

The resulting FASTQ files are processed and quantified using the GRCh38 human reference genome with the Cell Ranger single-cell toolkit (v.3.1.0). Quality control steps include filtering out: (1) low-quality cells expressing < 200 genes; (2) > 25,000 or < 500 UMIs; (3) Low-quality cells with > 20% mitochondrial gene count; (4) Cells judged as twin by DoubletFinder. All samples were integrated using the Seurat or Seurat-wrappers package (v0.1.0). Raw counts were normalized with a scale factor of 10,000 per cell and subsequently log-transformed (log [count+1]). The top 2000 highly variable genes (HVGs) were identified for integration purposes. Data scaling and principal component analysis (PCA) were conducted using default settings. Significant principal components (PCs) were selected based on the elbow plot of the standard deviations of the PCs and were utilized for neighbor detection, followed by Louvain clustering ^[25]^ and uniform manifold approximation and projection (UMAP).^[26]^ The same analytical pipeline was applied for the detection of sub-clusters.

### Spatial enhanced resolution omics-sequencing (stereo-seq)

The tissue was embedded in an OCT embedding agent (Sakura, 4583) and stored at -80°C. It was then sectioned into 10 μm thick slices using a cryotome (Leica, CM1950). Tissue structure was identified through H & E staining. Appropriately sized sections were placed on the Stereo-seq chip, and tissue integrity was assessed using ssDNA staining (Invitrogen, Q10212). Following the manufacturer’s protocol, chip permeation, RNA capture, reverse transcription, and cDNA library construction were completed.

Spatial transcriptome data were processed using Spaceranger (v.1.2.2). To achieve spatial localization of cell subsets on the spatial transcriptomics (ST) slides, we integrated the Stereo-seq data with single-cell RNA sequencing (scRNA-seq) data using the Seurat package. Seurat estimated the expression abundance of each cell subset at each spatial location by analyzing the similarity of expression patterns, thereby mapping the complete spatial distribution of cell types. These results are suitable for downstream analysis.

### GeoMx digital spatial profiling

A whole transcriptome atlas comprising 18,000 genes and a proteome atlas consisting of 570 proteins were generated using the GeoMx Digital Spatial Profiler (NanoString) on formalin-fixed, paraffin-embedded (FFPE) tissue sections. The slides were stained with morphological markers, including PanCK (epithelial cell marker), CD3 (lymphocyte marker), CD68 (macrophage marker), and DAPI (DNA marker). Based on these markers, 178 circular and polygonal regions of interest (ROIs), along with two negative controls, were selected for analysis.

Digital counts for each gene were standardized following quality control procedures. Protein digital counts were normalized using internal spike-in controls (ERCC) and the signal-to-noise ratio (SNR). DESeq-based differential expression analyses for both transcript and protein counts will include FDR control *via* the Benjamini–Hochberg method, reporting only targets with adjusted p-values < 0.05 as significant.

### Imaging mass cytometry

The paraffin-embedded tissues were sectioned into 4 μm slices and heated at 68°C for 1 h. The slices underwent dewaxing twice in xylene, followed by rehydration through a graded series of ethanol solutions at concentrations of 95%, 85%, and 75%. Antigen retrieval was conducted by heating the slices at 100°C for 30 min, followed by natural cooling. Subsequently, the slices were washed twice with PBS-TB for 5 min each and then blocked with SuperBlock for 30 min. After blocking, the slices were washed three additional times with PBS-TB and incubated overnight at 4°C with a metal-labeled antibody cocktail (Maxpar® X8 Kit). Post-incubation, the slices were washed and treated with 1.25 μmol/L Intercalator-Ir in PBS-TB at room temperature for 30 min, followed by washes with PBS-TB and distilled water. The prepared sections were then scanned using a Hyperion Imaging System to generate multiple images.

The IMC data processing involved four steps: overflow compensation, image denoising, contrast enhancement, and cell segmentation. The range of label expression was normalized to the 99th percentile for each channel, and batch effects were corrected using Harmony (v0.1.0). Clustering of cells was performed using Rphenograph (v0.99.1) with 100 nearest neighbors, and the cluster means were visualized as an annotated heat map.^[27]^ The K-means clustering method with (k = 15) was employed to identify cell neighborhoods (CNs) using a clustering window of the 20 nearest cells, based on Euclidean distance. Cell-cell interactions were analyzed using an arrangement assay facilitated by the imcRtools package (v1.0.2).^[28]^

### Minimal residual disease detection

Upon obtaining the region-specific exome sequencing data, we integrated the data from each region to design a personalized MRD panel. Our surveillance strategy includes up to 150 somatic mutations identified in each patient, along with 21 well-established cancer-related driver mutations. In cases where a large number of somatic mutations are detected, we prioritize screening clonal mutations over subclonal mutations. To ensure the sensitive detection of low-frequency mutations, we employ ultra-deep sequencing at a depth of 100,000×, using a minimum input of 10 ng of circulating tumor DNA (ctDNA). ctDNA-based MRD is dynamically monitored to evaluate the clinical significance of ctDNA in plasma for detecting low levels of residual disease.

## Data analyses

### Multi-omics data integration

For the CLEVER cohort, we implemented a multidimensional profiling strategy encompassing three complementary analytical axes: molecular stratification (genomic whole-exome sequencing, transcriptomic RNA-seq, and proteomic GeoMx DSP protein), resolution scaling (bulk tissue analysis, single-cell resolution, and spatial mapping), and cross-platform integration. This framework was operationalized through molecularly barcoded aliquots derived from identical tumor regions, ensuring cellular and spatial concordance across omics layers.

#### WES + RNA-seq

Leveraging multi-region WES data, we reconstructed phylogenetic trees for each tumor to delineate evolutionary trajectory patterns within the Chinese lung cancer cohort, employing maximum parsimony-based phylogenetic reconstruction with bootstrap validation (*n* = 1000 replicates). Concurrently, we deconvolute immune cell composition, intercellular communication networks, and spatially resolved microenvironmental niches of different evolutionary trajectory patterns by integrating bulk RNA-seq, single-cell RNA-seq, and spatial transcriptomic profiles.

#### ScRNA-seq + Spatial Transcriptomics

At present, there are two main methods for integrating scRNA-seq and spatial transcriptome data: Deconvolution and Mapping. Deconvolution aims to separate discrete cell subpopulations from the mixture of mRNA transcripts at each capture point based on single-cell data. The GeoMx DSP hybrid gene expression data based on ROIs can construct cell type-specific gene expression characteristics using single-cell data through the SPOTlight,^[29]^ and then use the regression model to infer the proportion of each cell type in the GeoMx ROI. Mapping can locate the specified cell subtypes based on scRNA onto spatial sections and lock onto specific ecological niches of tissues. We use Seurat v4 anchors to map single-cell clusters onto spatial coordinates, estimating cell-type proportions at each spot and ROI. This leverages canonical correlation analysis (CCA) to align modalities and transfers cell-type labels from scRNA-seq to Stereo-seq.

#### IMC + Spatial Transcriptomics

After cell segmentation and clustering in IMC images, we co-register IMC cell neighborhoods with spatial transcriptomics coordinates through image registration algorithms and probabilistic coordinate mapping. This enables precise superimposition of protein expression-defined tissue microenvironments onto transcriptomically annotated cellular architectures. For cross-modal feature integration, we implement graph neural network architectures^[30]^ (Graph Convolutional Networks or Graph Attention Networks) that jointly learn from protein expression gradients and transcriptomic profiles within spatially constrained cellular graphs. If protein and gene expression share common underlying factors (such as cell type or functional status), we can also make inferences through probabilistic models (such as Bayesian factor analysis).

#### Multi-omics Data Integration

To integrate multi-omics features spanning genomic, transcriptomic, and spatial proteomic dimensions, we implemented the Cluster-of-Clusters Analysis (COCA) algorithm-a framework previously established in The Cancer Genome Atlas (TCGA) for identifying pan-cancer molecular subtypes.^[31, 32, 33, 34]^ The analytical workflow proceeded through three key phases:

First, individual omics platform clusters were derived through unsupervised t-SNE embedding, generating platform-specific sample groupings. These discrete cluster assignments were subsequently transformed into binary indicator matrices, where rows represented samples and columns encoded binarized cluster memberships. Second, we performed consensus clustering on the concatenated multi-omics matrix using a dynamic feature weighting approach. The optimal number of stable subgroups was determined by maximizing the average silhouette width across k = 2 to k = 10 through Monte Carlo cross-validation (*n* = 1000 iterations). Finally, to quantify the relative contribution of each datatype to integrative classification, we systematically evaluated all pairwise combinations using the same COCA framework. Cross-datatype cluster concordance was assessed *via* Adjusted Rand Indices (ARIs), with values > 0.65 considered strong biological agreement.

### Machine learning model development and validation

The patients recruited in the first phase of this study will be randomly divided into training set, test set and validation set according to the ratio of 6: 2: 2. Considering the “events per variable” (EPV) rule, we apply the following steps to develop a robust recurrence prediction model. First, we will not treat all molecular features as separate predictors. Instead, we will compute summary metrics to reduce dimensionality (see section 5.1). Then Least Absolute Shrinkage and Selection Operator (LASSO) regression will be applied within the training subset to identify the most informative predictors, where the penalty parameter will be selected *via* grid search in nested 5-fold cross-validation. This procedure typically yields less than 9 variables, satisfying EPV≥10 given an expected 80 events (26.7% of the 300 patients).

All parameter tuning will be confined to the training set to avoid information leakage. Machine learning-based classifiers will be implemented on the training set with 1000 rounds of ten-fold cross validation. In each round, the training set will be split into 10 subsets (folds); in each fold, a model will be trained on nine of the subsets and then validated on the remaining subset to tune the hyperparameters. Then we will apply all obtained models to the test set and compare the performance measurements. The optimal method will be used to construct the final model. The tested algorithms included: adaptive boosting (AdaBoost), decision tree, logistic regression, linear support vector machine (Linear SVM), radial basis function kernel support vector machine (RBF SVM), naive Bayes, nearest neighbors, neural net, quadratic discriminant analysis (QDA), random forest, and extreme gradient boosting (XGBoost). All machine learning algorithms have a solid research foundation.^[35]^

We will assess C statistics (area under the receiver-operator characteristic curve [ROC-AUC]), calibration, and clinical utility (decision-curve analysis) in the validation and test set. Finally, we determined an algorithm for developing predictive models, which has been widely popularized in the machine learning community and has excellent scalability, high running speed and state-of-the-art accuracy. It has better average performance than other algorithms in tests. Therefore, it is chosen for further optimization. The model willed be externally and respectively validated on two additional cohorts of NSCLC patients from published database^[10,11]^ and subsequent phases of the CLEVER study to confirm reproducibility and generalizability. If necessary, we will recruit a multi-center prospective multi-region cohort from collaborating institutions of medical alliance for further verification.

### Statistical analysis

The Wilcoxon test is used to compare distribution differences in genomic and transcriptomic biomarkers between groups, while Fisher’s exact test is applied to compare clinical variables and the number of gene mutations between groups. All p-values are two-sided, with *P* < 0.05 considered statistically significant.

#### Missing data handling

Clinical and laboratory data will be monitored for completeness. Missing data will be addressed *via* multiple imputation using chained equations (MICE), incorporating baseline covariates, clinical outcomes, and molecular features. We have defined a maximum acceptable loss-to-follow-up rate of 10%. If the actual loss rate exceeds 10%, we will compare baseline characteristics of completers and non-completers to assess potential bias. Sensitivity analyses by comparing complete-case and imputed datasets will be reported to demonstrate the impact of missingness on key study findings.

#### Confounding bias correction

To control for baseline imbalances between the neoadjuvant and naïve cohorts, we will implement the three methods as follows: (1) Propensity Score Matching (PSM): A logistic regression–derived propensity score will be used for 1: 1 nearest-neighbor matching (caliper = 0.2), including age, sex, smoking status, clinical stage, and ECOG performance status. Balance will be checked *via* standardized mean differences (<0.1). (2) Multivariable Regression: DFS and OS will be modeled in the unmatched full cohort, adjusting for the same covariates to verify robustness. (3) Subgroup Analyses: Pre-specified stratified analyses by clinical stage (I *vs*. II–IIIA) and ECOG (0 *vs*. 1) will be performed. Interaction p values will assess heterogeneity of treatment effect.

## Discussion

Phylogenetic and heterogeneity analyses conducted on single-region sampling cohorts from Eastern populations have revealed several distinctions between Eastern and Western lung cancer populations.^[9]^ However, these analyses are limitations in comprehensively assessing tumor heterogeneity, as they are primarily based on bioinformatics methods to infer clonal and subclone mutation without validation in large, multi-sample intratumoral lung cancer cohorts. Multi-region sampling more accurately evaluates intratumoral heterogeneity and clonal evolution and reflects the molecular and immune mapping of the entire tumor.^[4,5,36]^

Since 2012, the TRACERx team has pioneered the exploration of intratumor heterogeneity and branched evolution, with the goal of elucidating tumor molecular evolution patterns and genomic instability characteristics.^[37]^ Over the past decade, they have developed a comprehensive framework of molecular evolution hypotheses and bioinformatic algorithms to address intratumoral heterogeneity and genomic instability during lung cancer progression. In 2017, using multi-region genomic data from 100 lung cancer patients, they identified intratumoral heterogeneity as a significant prognostic factor and proposed a genomic timeline categorizing molecular evolution events into early and late stages of tumor progression.^[4]^ By 2023, these initial findings were validated in a larger cohort of 421 cases using more advanced analytical algorithms.^[5]^ In recent years, studies employing a multi-region tumor sampling approach to investigate the evolutionary patterns of Chinese lung cancer have gradually emerged. However, these studies often face limitations due to small sample sizes and a lack of robust validation of bioinformatic algorithms.^[11]^ Hence, establishing a substantial multi-region sampling cohort within the Chinese population is vital for elucidating molecular evolution patterns unique to Chinese lung cancer patients. By collecting comprehensive genetic variation data from multiple tumor regions, we aim to decode the molecular evolution of Chinese lung cancer. The CLEVER study is expected to serve as a reference and foundation for understanding the drivers of impending metastatic events, thereby improving our ability to predict and manage disease progression.

The advent of immune checkpoint inhibitors (ICIs) has revolutionized the perioperative integrated treatment of NSCLCs patients, ushering in a new era.^[38, 39]^ Clinical studies have demonstrated that neoadjuvant immunotherapy enables approximately 30% of NSCLC patients to achieve major pathologic response (MPR) or even a pathological complete response (pCR).^[40, 41]^ Despite these encouraging results, a significant number of patients do not benefit from neoadjuvant immunotherapy. Understanding the response mechanisms of neoadjuvant therapy is crucial for enhancing its therapeutic efficacy in resectable NSCLCs. However, no studies have yet explored the response mechanism of neoadjuvant immune efficacy from the perspective of tumor heterogeneity and evolution. In this study, we will uncover distinct immune characteristics and spatial structures during the neoadjuvant immune response.

MRD has proven to be a significant predictor of prognosis and therapeutic efficacy in lung cancer. Our previous research demonstrated that the tumor-node-metastasis-blood (TNMB) classification system provides superior prognostic predictive power compared to the traditional TNM staging, particularly when deciding on adjuvant therapy.^[42]^ This advancement is enabled by a patient-specific Prognostic and Potential Therapeutic Marker Tracking (PROPHET) approach, which monitors plasma ctDNA-MRD derived from tumor cells.^[43]^ Furthermore, the CLEVER study has shown that multi-region sampling yields more comprehensive somatic mutation information than single-region sampling. By tracking mutations in a personalized MRD panel from baseline tumor samples, we can assess evolutionary significance and clonality, aiding in therapeutic decision-making. For patients undergoing neoadjuvant therapy, the clinical utility of ctDNA in evaluating treatment efficacy and assessing prognosis will be further explored through dynamic ctDNA changes.

The findings from this study could significantly contribute to the development of personalized treatment strategies for lung cancer patients. The CLEVER study lays the groundwork for a deeper understanding of the pathogenesis, progression, and evolutionary patterns of lung cancer. This enhanced understanding may lead to improvements in screening, diagnosis, treatment, and prevention strategies for lung cancer.
